# Household costs in the United States for accommodating functional impairments associated with Duchenne muscular dystrophy: results from a caregiver survey

**DOI:** 10.1186/s13023-025-03794-1

**Published:** 2025-06-12

**Authors:** Bryan Innis, John Jarvis, Taylor Renteria, Ivana Audhya

**Affiliations:** 1 Sarepta Therapeutics, Inc., Cambridge, MA USA; 2 Medicus Economics, Milton, MA USA

**Keywords:** Duchenne muscular dystrophy, Caregiver, Family, Impact, Economic burden, Indirect costs

## Abstract

**Background:**

Individuals with Duchenne muscular dystrophy (DMD) often require mobility aids and accessible homes and vehicles. Because these accommodations are not typically reimbursed by insurance, costs largely fall upon patients’ families. Additionally, families often incur high out-of-pocket costs for DMD-related health services and treatments. Research describing these expenses is important to understand the full financial cost associated with DMD. The objective of this study was to quantify household costs associated with DMD-related accommodations.

**Methods:**

A cross-sectional, retrospective online survey was conducted among United States-based family caregivers who provided informal (unpaid) care to ≥ 1 household member with DMD for ≥ 12 months and paid for ≥ 1 home/vehicle-related expense in the past 5 years. Participants reported costs, external financial support, and affordability for (1) home/vehicle purchases/modifications, (2) non-reimbursed medical equipment, (3) non-reimbursed health services or drugs, and (4) other household expenses. Costs were averaged over all respondents and among those incurring costs (conditional means). Average costs were stratified by ambulatory status, upper-limb impairment, insurance coverage type, home ownership status, and annual combined household income.

**Results:**

Among 90 caregivers, 94.4% were female, 90.0% were White, and 82.1% were the primary caregiver. The mean (standard deviation [SD]) age of individuals with DMD (*n* = 106) was 14.5 (5.3) years. Over half (59.4%) were reported as non-ambulatory, 23.6% as ambulatory, and 17.0% as transitioning from ambulatory to non-ambulatory. Average (SD) household costs over the past 5 years were $78,303 ($78,411) for home/vehicle expenses and $14,071 ($27,427) for medical equipment. Major home/vehicle expenses included purchasing/modifying a handicap-accessible vehicle (64% in past 5 years; conditional mean 5-year cost: $47,997), modifying home entrances (61%; $16,750), and modifying bathrooms (46%; $13,512). Powered wheelchairs were the leading driver of medical equipment costs (43%; $15,718). Annual recurring, non-reimbursed health service and drug costs were $13,642 (SD $41,792). Other recurring annual household expenses were $2593 (SD $4144). Household costs generally increased with more advanced disease progression. Households were able to pay for some expenses using external funding from the US government (e.g., Medicaid waivers) and non-government resources (e.g., charitable foundations, independently raised funds). However, many caregivers reported having to forgo purchases due to prohibitive costs.

**Conclusions:**

Households incur substantial costs to accommodate the functional impairments experienced by individuals with DMD.

**Supplementary Information:**

The online version contains supplementary material available at 10.1186/s13023-025-03794-1.

## Introduction

Duchenne muscular dystrophy (DMD) is a rare, progressive neuromuscular disease that presents substantial challenges for patients throughout their lives. DMD predominantly affects males, with symptoms beginning in early childhood as motor and developmental delays [1–3]. Muscle deterioration leads to loss of ambulation in late childhood, and continued progression leads to upper-limb impairment (e.g., inability to raise hand to mouth) as well as decreased respiratory function [1, 4, 5]. As a result of disease progression and associated complications, individuals with DMD experience premature mortality [1, 2]. While there is currently no cure for DMD, available treatment options may extend survival, delay physical function deterioration, and improve quality of life compared with natural history [3, 5–9].

Families of patients with DMD can experience a considerable caregiving impact. Because most individuals with DMD are living at home due to the pediatric nature of DMD, families are generally responsible for medical costs not reimbursable by health insurance, including provider office visits, professional caregiving, supportive therapy (e.g., physical therapy), and medications. These costs typically increase with disease progression, which necessitates more intensive interventions, such as spinal surgery for scoliosis or tracheostomy to enable ventilatory support [10–17]. Additionally, families often provide extensive unpaid caregiving, which can inhibit the caregiver’s educational or career ambitions and reduce paid work hours and leisure time [12, 17–26]. These indirect and intangible costs for DMD may be even higher than the medical cost burden [12, 14, 27]. Additional forms of family spillover, including social isolation and disruption to family life, may be substantial [26].

Families also incur extensive non-medical costs to accommodate DMD-related functional impairments. For example, they may need to modify their homes by widening doorways, modifying bathrooms, or installing an elevator, and some may consider moving into new, more accessible homes [19, 28]. Similarly, they may need to install powered ramps or wheelchair lifts in their cars or purchase handicap-accessible vans [19, 28]. These accommodations can improve daily life for the individual with DMD by facilitating their independence and averting injuries, particularly as the disease progresses [19, 28]. Because these expenses are generally not covered by health insurance, families bear the majority of the costs.

To better characterize the full financial impact of DMD, there is a need for detailed research regarding the costs borne by patients’ families [26, 29]. Several such studies have been conducted to date, measuring expenses at the household level [12, 13, 22, 25]. While these studies provide valuable insights, certain aspects of their cost-reporting methods may limit broader applications of the research findings. The present study aimed to build on this literature by producing detailed estimates of the costs incurred by households in the United States (US) to accommodate the progressive nature of DMD. From this prior research, authors anticipated that household costs to US caregivers would be substantial, but did not have pre-specified hypotheses as to how updated data on household costs would differ from previous studies.

## Methods

### Study design

A cross-sectional, retrospective primary research survey was administered online to US-based family-member caregivers of individuals with DMD. A de novo survey instrument was qualitatively and quantitatively tested to incorporate feedback from caregivers. Deidentified responses were summarized to produce detailed household-level cost estimates.

### Participant recruitment and study sample selection

Participants were recruited to complete the survey between June 2023 and September 2023 in collaboration with Rare Patient Voice, an organization with a panel of caregivers for individuals with DMD (https://rarepatientvoice.com/). Recruitment targeted a convenience sample of adult (aged ≥ 18 years) family-member caregivers for individuals with DMD (≥ 2 years of age). Eligible caregivers had provided informal (unpaid) care to ≥ 1 household member with DMD for ≥ 12 months and could provide estimates of DMD-related household costs. Within the past 5 years, caregivers had to have paid for home- or vehicle-related expenses in any of the following categories: moved to or built a new home, modified home entrance (e.g., ramp), modified bathroom, modified interior home doorway(s), purchased and/or modified a handicap-accessible vehicle, modified bedroom, modified kitchen, or installed elevator or lift. Caregivers were required to have resided in the US for ≥ 12 months and to be able to understand, speak, and read English. Participating caregivers provided informed consent prior to the survey. Given the convenience sample approach to recruitment, a response count was not pre-specified for the recruitment period. Because the study was non-interventional, it was exempted from institutional review.

### Survey components

#### Study sample characteristics

Caregivers provided information regarding demographic and clinical characteristics of the individual(s) with DMD for whom they provided care. Caregivers who provided care to ≥ 2 individuals with DMD provided data separately for the oldest and youngest individuals with DMD (see the Supplemental Methods for survey questions regarding ambulatory status and upper-limb impairment). In addition to household-level information, caregivers also provided their own demographics and details regarding the nature of their caregiving role and responsibilities (primary, coprimary, secondary).

#### Household costs

Participants were asked about items in four cost categories: (1) home and vehicle purchases or modifications, (2) non-reimbursed medical equipment purchases, (3) non-reimbursed health services or drug costs (e.g., long-term care, professional caregiving, supportive therapy, out-of-pocket healthcare visits and drugs), and (4) other household expenses (e.g., adaptive clothing/shoes, incontinence products, non-pharmaceutical supplements/vitamins). The first two categories included expenses expected to be incurred infrequently or only once; consequently, participants were asked about purchases or payments made in the past 5 years. The third and fourth categories included items more likely to be purchased or paid for on a repeated basis; therefore, participants were asked about purchases or payments in the past 3 months. For each item, participants were asked whether their household incurred a cost in the relevant period. For each item for which a purchase or payment was reported, participants were asked to select from a category or cost range corresponding to the total amount paid by their household during the relevant period. Cost ranges were determined based on discussions with caregivers during survey piloting. Additionally, participants were asked whether their household had been unable to make a purchase or payment due to prohibitive costs.

Participants were also asked about any external financial resources they used to pay for each item. They were asked what percentage of the item’s total cost was paid for out of pocket, what percentage was paid for using government resources (e.g., a Medicaid waiver), and what percentage was paid for using non-government external resources (e.g., private health insurance, charitable foundations, community groups, other family members or friends, or raised funds).

### Statistical analysis

#### Study sample characteristics

Characteristics of individuals with DMD, caregivers, and households were summarized descriptively. Frequencies and percentages were used to summarize categorical variables, while means and standard deviations (SDs) were used to summarize continuous variables.

#### Household expenses

The proportion of households that incurred each expense was reported, as well as the proportion of households unable to make a purchase or payment due to prohibitive costs.

Mean (SD) household cost was calculated for each item. For home or vehicle purchases or modifications and non-reimbursed medical equipment purchases, costs were summarized as reported (i.e., over 5 years) without adjustment for inflation. For items related to non-reimbursed health services and drugs or other household expenses, costs were annualized based on the costs reported for the past 3 months. Conditional means (SDs) and medians (interquartile ranges [IQRs]), calculated as the average or median costs incurred only among households that reported an expense, are also reported.

To enable estimation of means (SDs) and medians (IQRs) for costs, responses provided in the form of cost ranges were assumed to equal the midpoint value of the selected range. For example, if a participant indicated that their household spent $1000 to $2499 on modifying bathrooms in the past 5 years, a cost of $1749.50 was assumed. If “$150,000 or higher” was selected, the cost was assumed to equal $150,000.

Sources of external financial support were descriptively assessed. For each item, the percentage of total household costs that was paid for using each financial resource was averaged across all households that incurred costs.

#### Subgroup analyses

Mean (SD) costs were calculated for key subgroups. For one stratification of interest, participants were grouped by the caregiver-reported ambulatory status of the individual with DMD. In the event that a household had two or more individuals, the stratification was by the most progressed ambulatory stage. For another subgroup analysis, participants were grouped by the caregiver-reported upper-limb impairment of the individual with DMD. In the event that a household had two or more individuals, the stratification was by the most impaired upper-limb function. Results were also stratified by household according to health insurance type for individuals with DMD in their household (incorporating the following hierarchy): (1) whether any individual with DMD in their household had both employer-sponsored and state-level health insurance (e.g., Medicaid); (2) whether any individual with DMD in their household had state-level health insurance only; and (3) whether any individual with DMD in their household had employer-sponsored health insurance only. Finally, results were stratified by household home ownership status (i.e., home owners vs. non-home owners) and annual combined household income (i.e., annual combined household income less than $100,000 vs. greater than or equal to $100,000).

## Results

### Study sample

The survey yielded responses from 90 caregivers from distinct households representing 106 individuals with DMD. Among the 106 individuals with DMD, the mean (SD) age was 14.5 (5.3) years, with a mean (SD) time since diagnosis of 10.4 (5.4) years (Table 1). Nearly all individuals had both medical and pharmacy coverage during the prior 12 months, with the majority covered through employer-sponsored health plans (67.9%) and/or state health insurance (58.5%) (Supplemental Table S1). Most (59.4%) individuals with DMD were non-ambulatory; 23.6% were ambulatory and 17.0% were transitional. In terms of upper-limb function, 37.7% had no impairment, 36.8% had mild impairment, and 25.5% had moderate-to-severe impairment. The highest reported comorbidities were anxiety, learning disabilities, scoliosis, attention-deficit/hyperactivity disorder, and osteoporosis.

**Table 1:** Characteristics of individuals with DMD and their caregivers

Patient demographic characteristics	*N* = 106
Age, years, mean (SD)	14.5 (5.3)
Age, years, n (%)	
2–5	4 (3.8)
6–12	35 (33.0)
13–17	41 (38.7)
18+	26 (24.5)
Sex, n (%)	
Female	0 (0.0)
Male	106 (100.0)
Race, n (%)^a^	
American Indian or Alaska Native	3 (2.8)
Asian	6 (5.7)
Black or African American	5 (4.7)
Hispanic or Latino	11 (10.4)
White	92 (86.8)
Other^b^	1 (0.9)
**DMD-related characteristics**	
Age at DMD diagnosis, years, mean (SD)^c^	4.1 (2.5)
Years since DMD diagnosis, mean (SD)^c^	10.4 (5.4)
Ambulatory status, n (%)	
Ambulatory	25 (23.6)
Transitional	18 (17.0)
Non-ambulatory	63 (59.4)
Upper-limb function, n (%)	
No impairment	40 (37.7)
Mild impairment	39 (36.8)
Moderate impairment	24 (22.6)
Severe impairment	3 (2.8)
**Caregiver demographic characteristics**	***N*** ** = 90**
Age, years, mean (SD)^c^	45.6 (8.4)
Age, years, n (%)	
0–17	0 (0.0)
18–39	23 (25.6)
40–64	65 (72.2)
65+	1 (1.1)
Missing age	1 (1.1)
Sex, n (%)	
Female	85 (94.4)
Male	5 (5.6)
Race, n (%)^c^	
American Indian or Alaska Native	2 (2.2)
Asian	1 (1.1)
Black or African American	2 (2.2)
Hispanic or Latino	7 (7.8)
White	81 (90.0)
Other^b^	0 (0.0)
Total combined household income last year, n (%)^c^	
$0 to $49,999	15 (16.7)
$50,000 to $99,999	26 (28.9)
$100,000 and higher	45 (50.0)
Other	4 (4.4)
**Caregiving-related characteristics**	
Number of individuals with DMD receiving care, n (%)	
1	74 (82.2)
2+	16 (17.8)
Relationship of the survey respondent to the individual(s) with DMD, n (%)^d^	
Mother	98 (92.5)
Father	5 (4.7)
Other	3 (2.8)
Caregiver responsibilities, n (%)^d^	
Primary caregiver	87 (82.1)
Secondary caregiver	2 (1.9)
Evenly splits responsibilities with another household member	17 (16.0)
Living arrangement for individual(s) with DMD, n (%)^d^	
Live most days and nights of the week in their household	105 (99.1)
Do not live most days and nights of the week in their household	1 (0.9)

DMD, Duchenne muscular dystrophy; SD, standard deviation

^a^Participants could select multiple response categories. Therefore, percentages may sum to over 100%

^b^“Other” responses included options for “Other,” “Prefer not to answer,” or “Unsure”

^c^Calculated among all non-missing survey responses

^d^Responses sum to the total number of DMD individuals represented in survey results

Among the 90 caregivers, the mean (SD) caregiver age was 45.6 (8.4) years (Table 1). Most caregivers were female, mothers of patients with DMD, White, and married or living with someone. Most (57.8%) had a Bachelor’s degree or higher; 40.0% were employed full-time, and 16.7% were employed part-time at the time of the survey (Supplemental Table S1). Most (82.1%) considered themselves the primary caregiver, and 16.0% reported evenly splitting caregiving responsibilities.

The most prevalent household income levels were between $50,000 to $99,999 (28.9% of households) and between $100,000 to $149,999 (30.0%), and most were in either the Midwest or South and in a town or suburban area (Supplemental Table S1). Most households (76.7%) resided in properties that were owned or co-owned and had one (41.1%) or two (48.9%) members working for pay in the past 12 months.

### Household costs

**Fig. 1:**
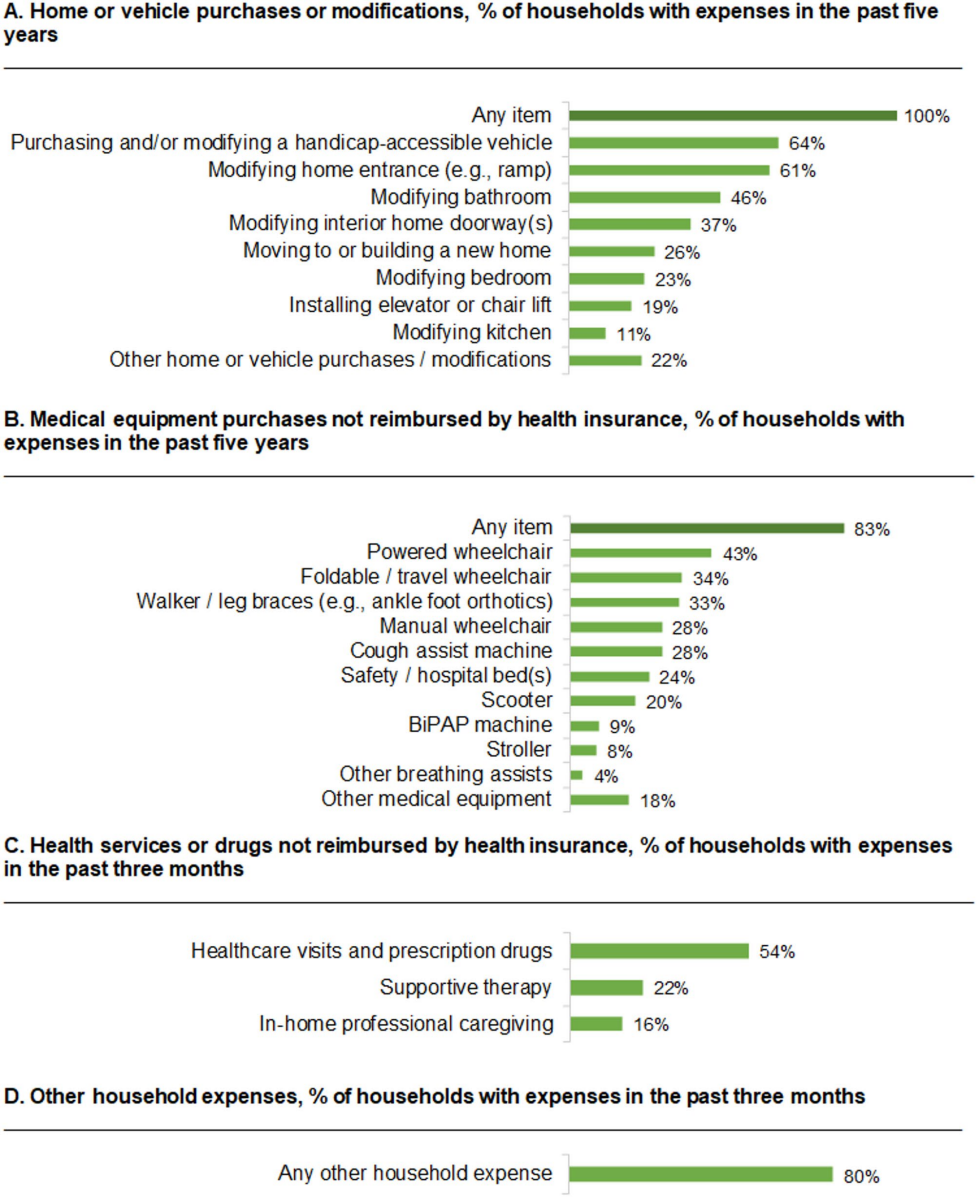
Proportion of households incurring costs (*N* = 90). BiPAP, bilevel positive airway pressure

All 90 households incurred at least one expense in the past 5 years related to home or vehicle purchases or modifications, as was required for study inclusion (Fig. 1). The most common costs incurred were related to purchasing and/or modifying a handicap-accessible vehicle, modifying home entrances (e.g., ramp), modifying bathrooms, and modifying interior home doorways. Most households (83%) reported at least one medical equipment purchase not reimbursed by health insurance in the past 5 years. The most common items purchased in the past 5 years were powered wheelchairs, foldable/travel wheelchairs, walker/leg braces (e.g., ankle foot orthotics), manual wheelchairs, and cough assist machines. Just over half of households (54%) made non-reimbursed payments for healthcare visits or prescription drugs in the past 3 months, 22% made payments for supportive therapy, and 16% made payments for in-home professional caregiving.

Across all households, $78,303 was spent over 5 years on home and vehicle purchases or modifications (Table 2). The leading contributor was purchasing and/or modifying a handicap-accessible vehicle, which had a mean cost of $30,932. Moving to or building a new home was the second most expensive item, with a mean cost of $19,679. Households also spent a mean of $10,236 in modifying home entrances. Medical equipment purchases cost an average of $14,071 over 5 years. With respect to non-reimbursed payments for health services or drugs, households paid a total of $13,642 each year out of pocket. The most expensive reported item was healthcare visits and prescription drugs, with a mean annualized cost of $8184. In-home professional caregiving had an average cost of $4529 per year, and supportive therapy costs were an average of $929. Conditional mean (SD) and median (IQR) costs among households that incurred expenses are also reported in Table 2 and Supplemental Table S2, respectively.

**Table 2:** Average household expenses

	Overall average^a, b^	Conditional average^b, c^	
	Mean (SD)	Households that incurred costs, *n* (% of all households)	Conditional mean (SD) costs
**Home or vehicle purchases or modifications (5-year cost)**	$78,303 ($78,411)	90 (100)	-
Moved to or built a new home	$19,679 ($47,344)	23 (26)	$77,005 ($66,729)
Modified home entrances (e.g., ramp)	$10,236 ($23,697)	55 (61)	$16,750 ($29,081)
Modified bathroom	$6155 ($12,525)	41 (46)	$13,512 ($15,722)
Modified interior home doorway(s)	$991 ($2122)	33 (37)	$2704 ($2783)
Purchased and/or modified a handicap-accessible vehicle	$30,932 ($33,034)	58 (64)	$47,997 ($29,765)
Purchased a new vehicle		17 (19)	$65,441 ($19,966)
Purchased a used vehicle		39 (43)	$42,403 ($26,896)
Other		7 (8)	$8035 ($12,819)
Modified bedroom	$1192 ($4155)	21 (23)	$5107 ($7472)
Modified kitchen	$1889 ($6961)	10 (11)	$17,000 ($13,922)
Installed elevator or lift	$5289 ($18,270)	17 (19)	$28,000 ($36,739)
Elevator		5 (6)	$22,650 ($14,289)
Stair lift		6 (7)	$5175 ($4895)
Platform lift		3 (3)	$28,333 ($5774)
Pool lift		4 (4)	$10,833 ($2887)
Ceiling track		7 (8)	$14,714 ($12,044)
Patient lift (e.g., Hoyer lift, Molift)		6 (7)	$3375 ($4488)
Other home or vehicle purchases or modifications	$1940 ($6062)	20 (22)	$8732 ($12,663)
**Medical equipment purchases not reimbursed by health insurance (5-year cost)**	$14,071 ($27,427)	75 (83)	$16,885 ($30,309)
Scooter		18 (20)	$3110 ($4184)
Stroller		7 (8)	$708 ($813)
Powered wheelchair		39 (43)	$15,718 ($21,979)
Manual wheelchair		25 (28)	$3837 ($8485)
Foldable/travel wheelchair		31 (34)	$2084 ($1804)
Walker/leg braces (e.g., ankle foot orthotics)		30 (33)	$1298 ($1830)
Safety/hospital bed(s)		22 (24)	$3513 ($4431)
BiPAP machine		8 (9)	$437 ($651)
Cough assist machine		25 (28)	$967 ($1332)
Other breathing assists		4 (4)	$1937 ($2563)
Other medical equipment		16 (18)	$4278 ($7741)
**Health services or drugs not reimbursed by health insurance (1-year cost**,** annualized)**	$13,642 ($41,792)		
In-home professional caregiving	$4529 ($20,115)	14 (16)	$29,113 ($46,538)
Supportive therapy	$929 ($2401)	20 (22)	$4182 ($3660)
Healthcare visits and prescription drugs	$8184 ($37,124)	49 (54)	$15,031 ($51,701)
**Other expenses (1-year cost**,** annualized)**	$2593 ($4144)	72 (80)	$3241 ($4404)

BiPAP, bilevel positive airway pressure; SD, standard deviation

^a^Calculated among all households represented in the study sample (*N* = 90)

^b^5-year costs are presented for items related to home or vehicle purchases or modifications and medical equipment purchases not reimbursed by health insurance. 1-year, annualized costs are presented for health services or drugs not reimbursed by health insurance. 1-year, annualized costs are presented for other expenses

^c^Calculated among households that incurred costs and provided a corresponding cost estimate

#### Forgone expenses due to prohibitive costs

Multiple home- or vehicle-related expenses were commonly forgone due to being cost prohibitive (Supplemental Fig. S1). Most frequently reported were moving to or building a new home (30%), modifying bathrooms (29%), and purchasing and/or modifying a handicap-accessible vehicle (28%). Households were unable to purchase some medical equipment due to costs, including foldable/travel wheelchairs (17%), powered wheelchairs (9%), scooters (7%), and safety/hospital beds (7%).

#### Financial resources used to pay for household expenses

**Fig. 2:**
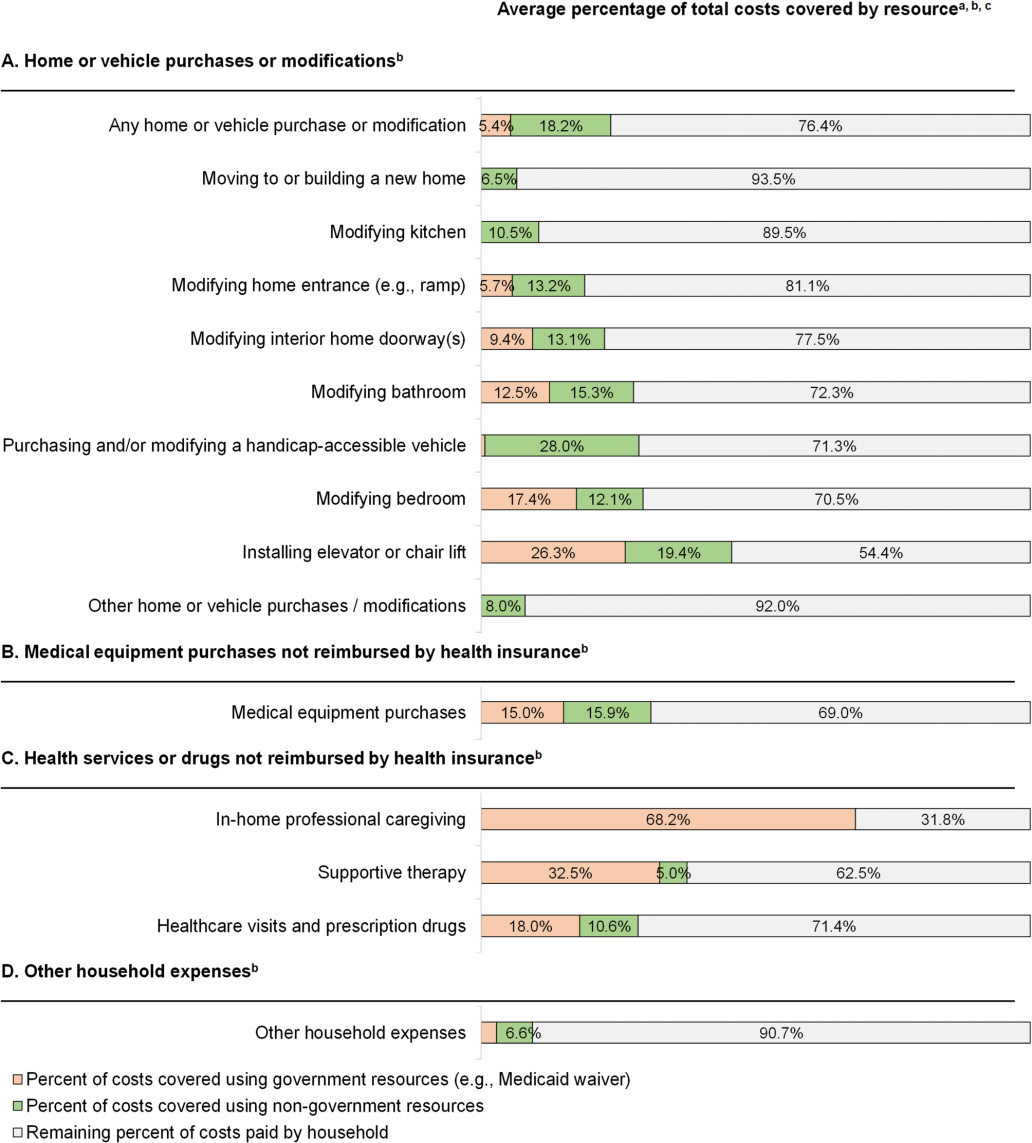
Financial resources used to pay for household expenses^**a−c**^. ^**a**^For each item, the mean share of expenses paid by each resource was calculated across all households who incurred an expense. ^**b**^Non-government resources include private health insurance, charitable foundations, community groups, other family members or friends, raised funds, or other sources. For “Medical equipment purchases not reimbursed by health insurance” and “Health services and drugs not reimbursed by health insurance,” non-government resources do not include private health insurance. ^**c**^Rows may not sum to 100%, as reported values are rounded to one decimal place

On average, households paid for 76.4% of their total home and vehicle expenses out of pocket and relied on external financial resources to pay for the remaining (Fig. 2). Government resources on average covered 5.4% of these expenses, while non-government resources, such as charitable foundations or raised funds, covered 18.2%. Government and non-government resources were used to pay for 15.0% and 15.9% of medical equipment purchases, respectively.

### Subgroup analyses

#### Ambulatory status

**Fig. 3:**
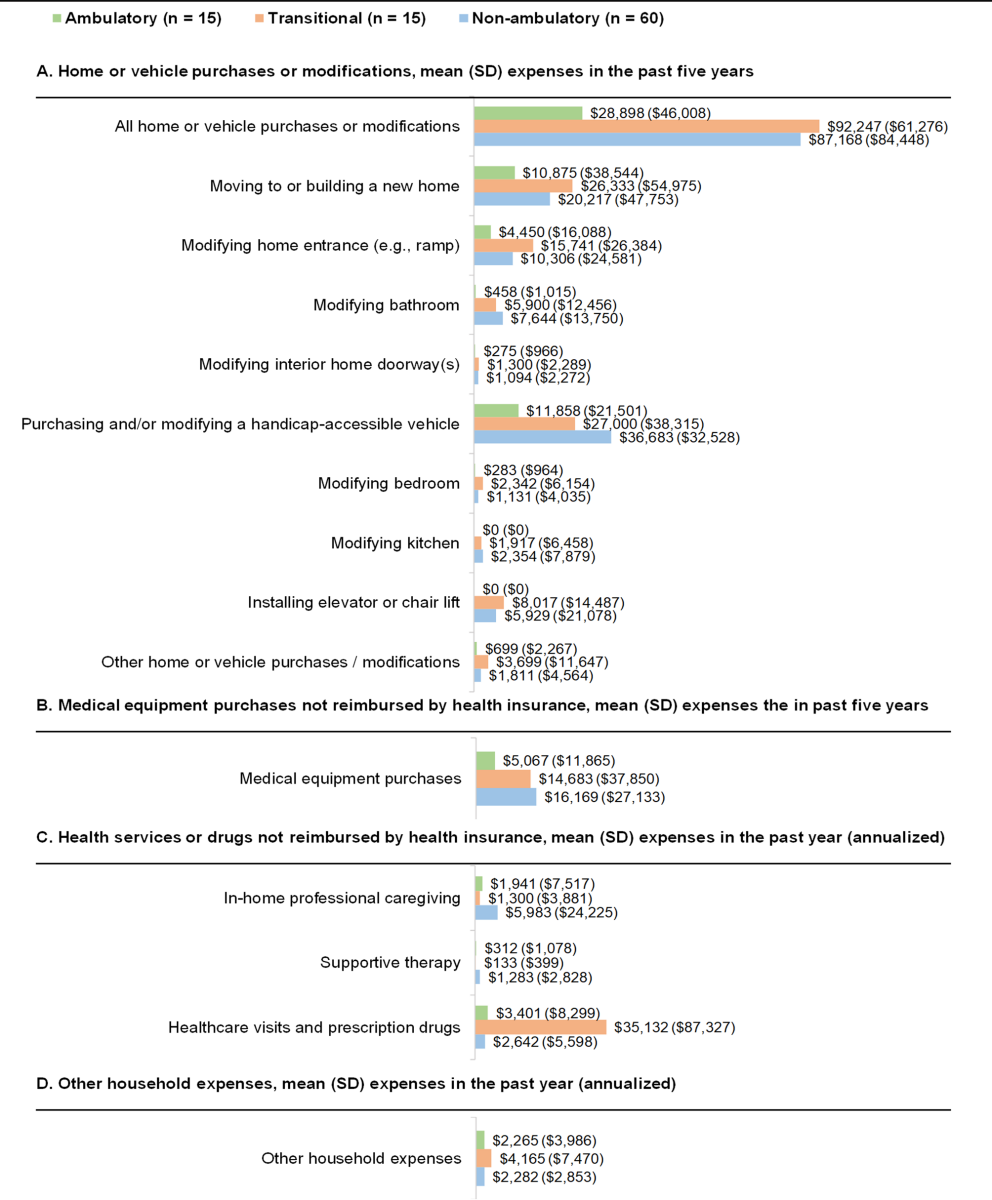
Average costs, stratified by ambulatory status. Note: mean (SD) costs were calculated among all households in each subgroup. SD, standard deviation

Certain household expenses differed by ambulatory status, although differences between households caring for patients with transitional status and non-ambulatory status were not consistent. For instance, households were more likely to purchase or modify a handicap-accessible vehicle in the past 5 years as the individual with DMD progressed (ambulatory, 33%; transitional, 47%; non-ambulatory, 77%) (Supplemental Fig. S2). A similar relationship was observed for medical equipment purchases and healthcare visits and prescription drugs. The overall mean cost of home- and vehicle-related purchases was approximately three times higher in the transitional subgroup and the non-ambulatory subgroup than in the ambulatory subgroup ($92,247 and $87,168 vs. $28,898, respectively). Similarly, the mean cost of medical equipment was approximately three times higher in the transitional subgroup and the non-ambulatory subgroup than in the ambulatory subgroup ($14,683 and $16,169 vs. $5067, respectively) (Fig. 3).

#### Upper-limb impairment

Similar trends were observed based on upper-limb impairment. Households were more likely to purchase or modify a handicap-accessible vehicle in the past 5 years as upper-limb impairment increased (no impairment, 48%; mild impairment, 66%; moderate-to-severe impairment, 80%), as well as to incur expenses for other home modifications (Supplemental Fig. S3). One notable exception was the likelihood of moving to or building a new home in the past 5 years, which decreased along with upper-limb impairment (no impairment, 52%; mild impairment, 16%; moderate-to-severe impairment, 12%). Average home- and vehicle-related costs over the past 5 years were consistent across upper-limb impairment subgroups, although costs to purchase or modify a handicap-accessible vehicle were higher in households with more severe upper-limb impairment and costs to move or build a new home were lower in households with more severe upper-limb impairment (Supplemental Fig. S4). Household costs for medical equipment and in-home professional caregiving also increased with greater upper-limb impairment.

#### Other subgroups

Subgroup results were also generated based on insurance coverage type represented across households (Supplemental Fig. S5; Supplemental Fig. S6), home ownership (Supplemental Fig. S7; Supplemental Fig. S8), and annual combined household income (Supplemental Fig. S9; Supplemental Fig. S10). Costs were generally higher for households with employer-sponsored insurance coverage only, compared with households with both employer-sponsored and state-level insurance or state-level insurance only. In general, costs were also higher for households that owned their own home or had annual combined incomes of $100,000 or higher.

## Discussion

The present study reports detailed estimates of out-of-pocket costs among a sample of households predominantly composed of individuals with higher socioeconomic status that incurred DMD-related home or vehicle expenditures within the past 5 years. These estimates are important for informing the societal costs of DMD, particularly for individuals who have experienced disease progression. However, for a full societal cost analysis, additional data on the proportion of households incurring DMD-related home and vehicle costs would be required. The findings also shed light on the need for additional financial support for families caring for individuals with DMD.

Over a 5-year period, households experienced substantial variability in DMD-related expenses, reflecting differences in disease progression, expenditure categories, and annual household incomes. For example, average 5-year costs for home and vehicle modifications were lowest for households caring for ambulatory individuals with DMD (*n* = 15; $28,898), non-homeowners (*n* = 21; $42,886), households earning less than $100,000 per year (*n* = 41; $60,735), and households with state-level insurance coverage only (*n* = 26; $47,319). Conversely, average 5-year costs were highest for households caring for transitional (*n* = 15; $92,247) or non-ambulatory individuals with DMD (*n* = 60; $87,168), homeowners (*n* = 69; $89,082), households earning $100,000 per year or more (*n* = 45; $94,881), and households with employer-sponsored insurance coverage only (*n* = 34; $109,218). Trends in costs generally followed the needs of individuals with DMD: as ambulatory function decreases, household expenditures increase. For many items, costs were several times higher in households in which at least one individual with DMD was either transitional/non-ambulatory or had moderate-to-severe upper-limb impairment. These findings may suggest an uneven financial burden faced by families, highlighting a gap where those with fewer resources might struggle to meet the necessary costs.

Households reported to be able to pay for some of their expenses using external funding from the government (e.g., Medicaid waivers), as well as from non-government resources, such as charitable foundations, family members and friends, and independently raised funds. Nonetheless, many caregivers reported that the households bore the majority of the costs and that prohibitive costs had forced their households to forgo purchases for needed items in a variety of categories.

The present study builds on an existing body of literature regarding non-medical resource use and costs related to DMD. In a recent online survey of US family caregivers for children with DMD, Schwartz et al. [19] reported the frequency of various household accommodations as well as purchases and modifications of handicap-accessible vans. Although the present study observed a higher rate of vehicle-related purchases and/or modifications (64% vs. 45% in Schwartz et al. [19]), rates of specific home modifications were generally similar across the two studies. In contrast to the present study, it should be noted that expenditures reported in Schwartz et al. [19] included all previous purchases (not limited to the past 5 years) and the survey sample was not restricted to households incurring DMD-related home or vehicle expenditures within the past 5 years.

With respect to studies presenting cost estimates, differences in methods limit our ability to directly compare findings. Work by the Lewin Group [13, 25] found that households incurred annual DMD-related costs of $3050 to move or modify a home, $1680 to purchase or modify a motor vehicle, and $3189 for professional caregiving (2010 USD). While some results from our study are comparable to these findings, such as 5-year costs for moving to or building a new home, results from our study found higher 5-year costs for vehicle-related expenses and professional caregiving. While the drivers of cost differences between the two studies cannot be definitively ascertained, the recall period used to calculate annual costs may be a factor. Whereas the present study asked about costs incurred in the past 5 years or 3 months, the Lewin Group study [13, 25] asked about cumulative costs over the lifetime of individuals with DMD and annualized these costs since the date of DMD diagnosis. Similar to Schwartz et al. [19], the study sample was also not restricted to households that incurred DMD-related home or vehicle costs in the past 5 years.

Landfeldt et al. [12] estimated that households incurred approximately $7930 (2012 USD) per year in costs related to aids, devices, and investments, including home modifications (e.g., adaptations for wheelchair accessibility). Results from the present study showed higher implied annual costs for home and vehicle expenses, although it is unclear which specific cost items were included in the Landfeldt et al. [12] estimate. As with the Lewin report [13, 25], differences in study methods prevent a conclusive comparison of annual cost estimates. For instance, Landfeldt et al. [12] used a 1-year recall period, which may lead to the exclusion of expenses typically incurred only once or every few years (e.g., home modifications or vehicle-related expenses). Additionally, while Landfeldt et al. [12] noted that country-specific cost estimates for aids, devices, and investments were obtained through expert input, limited additional information was provided. Finally, similar to Schwartz et al. [19] and the Lewin report [13, 25], the study sample in Landfeldt et al. [12] was not restricted to households that incurred DMD-related home or vehicle costs in the past 5 years.

### Limitations

Our findings are subject to several limitations inherent in the design of any cross-sectional, single-cohort survey. First, the findings must be interpreted as associational rather than causal. Second, due to the cross-sectional design of the survey, it was not possible to track changes in costs for individual households over time as individuals with DMD progressed through the disease. Third, because this study used retrospective, self-reported data, cost estimates are subject to recall bias. To address this, the present study intentionally recruited caregivers who had incurred relevant costs in the past 5 years, and survey questions were focused on costs incurred within this time frame. As a result, our study sample does not include households without DMD-related home or vehicle expenses in the past 5 years. Thus, this study did not capture data from households that had expenses in categories 2, 3, and/or 4 only or those households that had to forgo an expense because they could not afford it. This recruitment approach may have led to an under-representation in the study sample of households caring for individuals with DMD in less advanced disease stages. To illustrate how costs vary across the patient and family experience, study results were separately stratified and reported by disease stage across two dimensions: ambulatory status and upper-limb impairment. Fourth, cost estimates are based on the center point of range of values, and as such do not reflect exact costs for the various categories.

Findings from this study may also not be generalizable to all US households caring for individuals with DMD. For instance, Asian, Black or African American, and Hispanic or Latino caregivers comprised 1%, 2%, and 8% of the study sample, but represent approximately 6%, 14%, and 20% of the overall US population [30]. Additionally, educational attainment and household income in the study sample differed from the US population. Most notably, a higher proportion of the sample had a Bachelor’s degree (37%) than the US adult population (22%) [31]. Similarly, caregivers in the sample reported higher household incomes than the general US population: 30% of the sample reported an annual household income of $100,000 to $149,999 versus 17% of estimated US households, and 17% of the sample reported an income less than $49,999 compared to an estimated 31% of US households [32]. Further research should aim to explore whether disparities in these or other sociodemographic characteristics influence the household costs and financial strain borne by families caring for individuals with DMD.

Finally, the present study was focused on quantifying one component of economic impact related to DMD: household costs to accommodate care needs for individuals with DMD. It should be noted, however, that a full assessment of the US economic impacts from DMD would need to account for a broader set of perspectives across numerous stakeholders, including health insurance payers, governing bodies at the federal and state level, and individuals with DMD. Even among caregivers for individuals with DMD, critical areas of economic impact such as employment/work productivity and leisure time loss were not quantified in the present study due to survey time constraints. Future research focused on these additional perspectives is warranted to gain a more complete understanding of the economic impacts related to DMD.

## Conclusions

Families with individuals with DMD face a range of financial challenges and substantial household costs as they seek to provide the necessary support and accommodations for their loved ones. Costs were particularly high in households caring for individuals with more advanced disease and limited ambulatory function. This research provides additional detail on both infrequent and recurring costs for these households, as well as the rate that such expenses are forgone due to affordability barriers. The study findings provide additional insight into the significant financial impact that DMD poses on caregivers and households.

## Electronic supplementary material

Below is the link to the electronic supplementary material.

Supplementary Material 1

Supplementary Material 2

Supplementary Material 3

Supplementary Material 4

Supplementary Material 5

Supplementary Material 6

Supplementary Material 7

Supplementary Material 8

Supplementary Material 9

Supplementary Material 10

Supplementary Material 11

Supplementary Material 12

Supplementary Material 13

## Data Availability

Data used for this study are not available due to participant privacy protections.

## Abbreviations

ADHD: Attention deficit/hyperactivity disorder
BiPAP: Bilevel positive airway pressure
CHAMPVA: Civilian Health and Medical Program of the Department of Veterans Affairs
CPAP: Continuous positive airway pressure
DMD: Duchenne muscular dystrophy
GED: General Educational Development
IHSS: In-home supportive services
IQR: Interquartile range
OOP: Out-of-pocket
SD: Standard deviation
SCHIP: State Children’s Health Insurance Program
VA: Veterans’ Affairs
US: United States
USD: US dollars
