# Supplementary figures and images for: Household costs in the United States for accommodating functional impairments associated with Duchenne muscular dystrophy: results from a caregiver survey
Source: Orphanet J Rare Dis. 2025 Jun 12;20:301. doi: 10.1186/s13023-025-03794-1 (PMC12160368; doi:10.1186/s13023-025-03794-1)

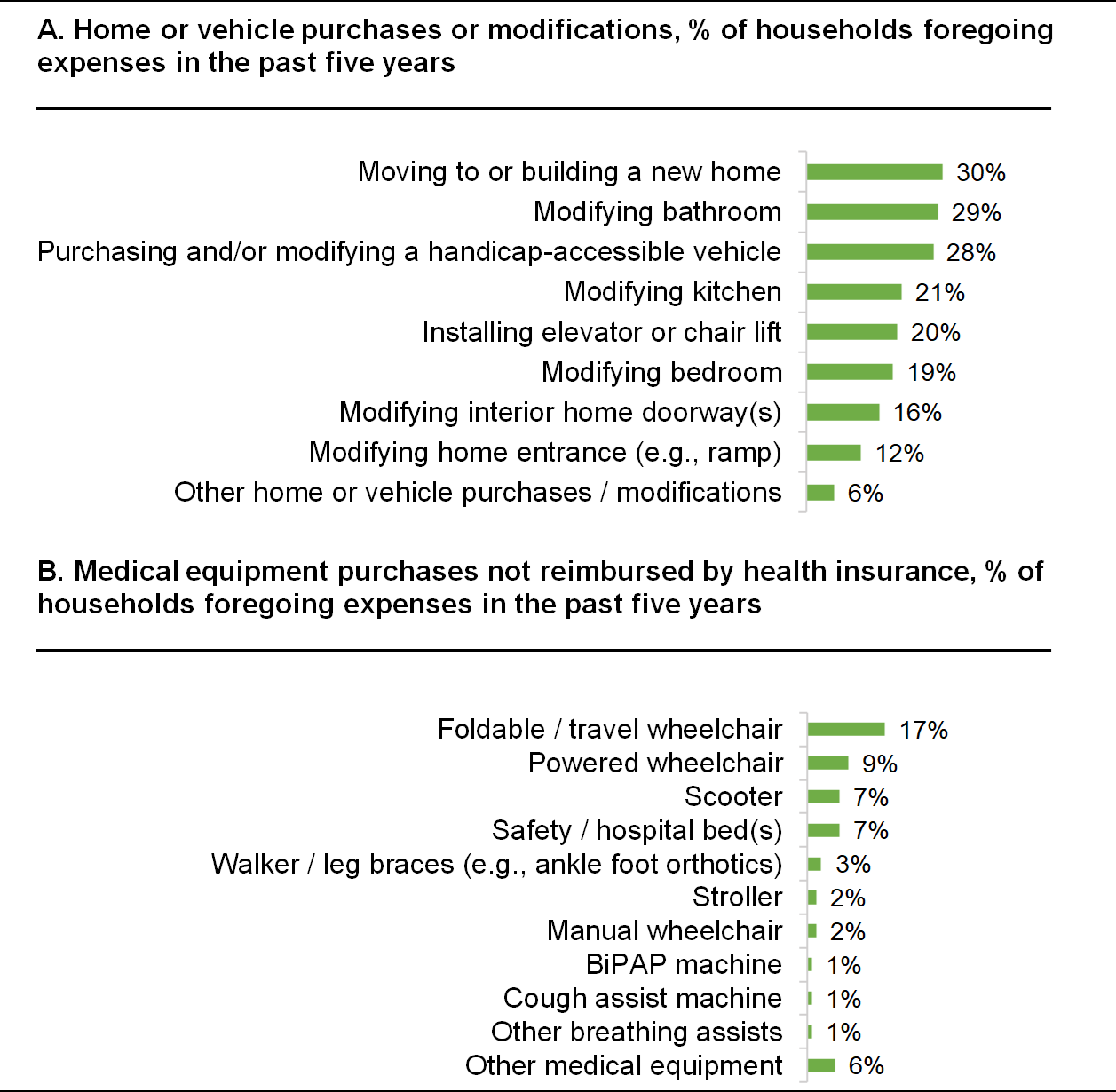

Supplement: Supplementary file 1 — Supplementary Material 1 [file 13023_2025_3794_MOESM1_ESM.png]

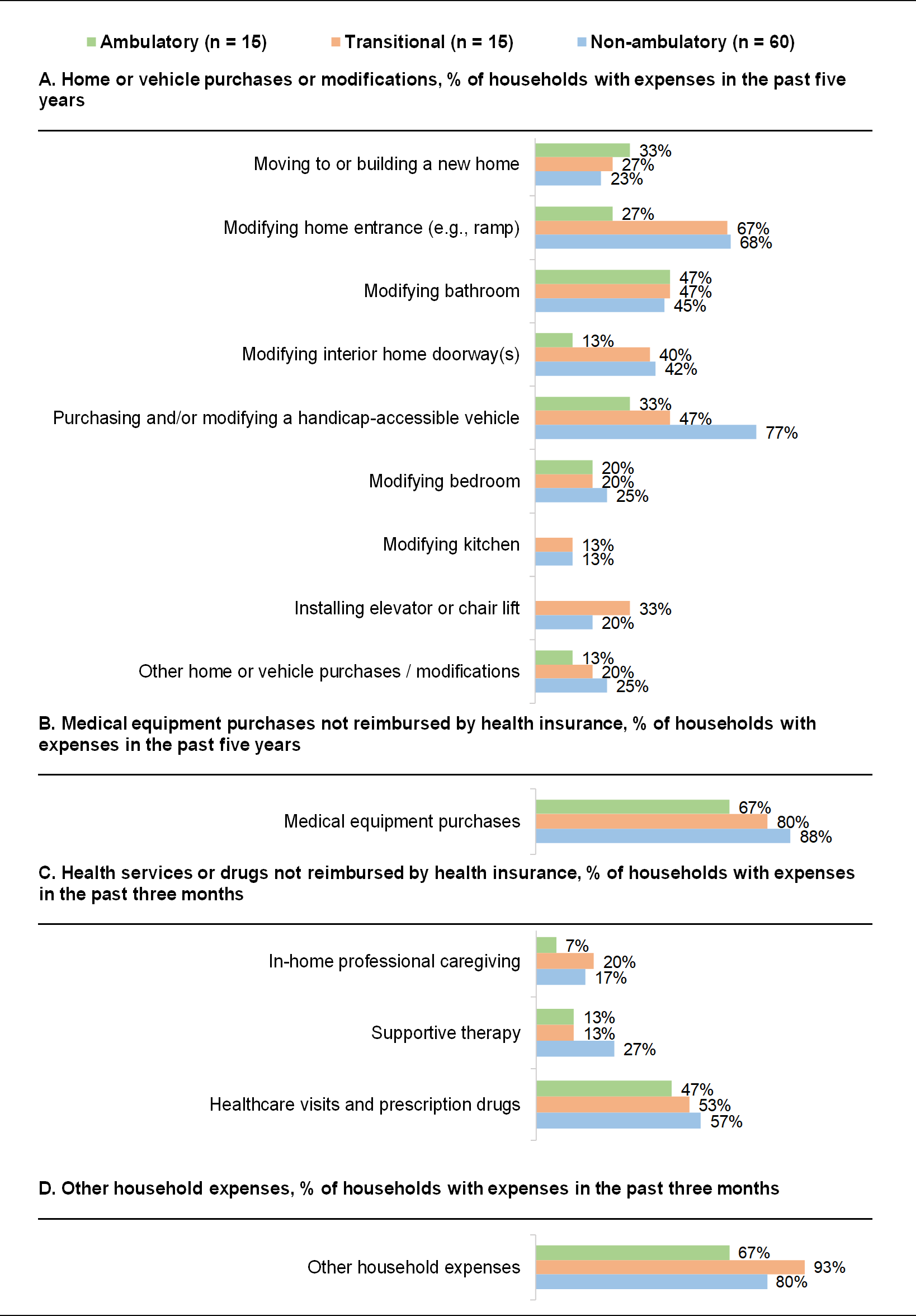

Supplement: Supplementary file 2 — Supplementary Material 2 [file 13023_2025_3794_MOESM2_ESM.png]

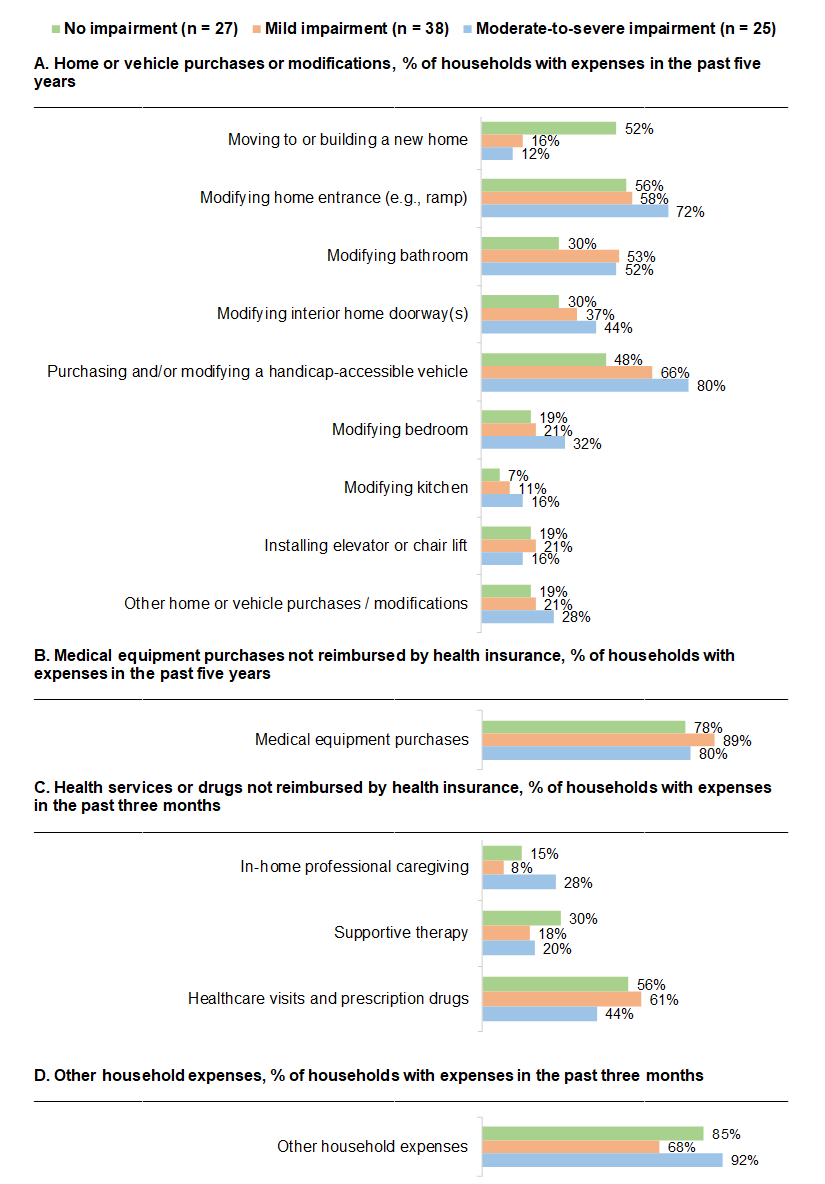

Supplement: Supplementary file 3 — Supplementary Material 3 [file 13023_2025_3794_MOESM3_ESM.png]

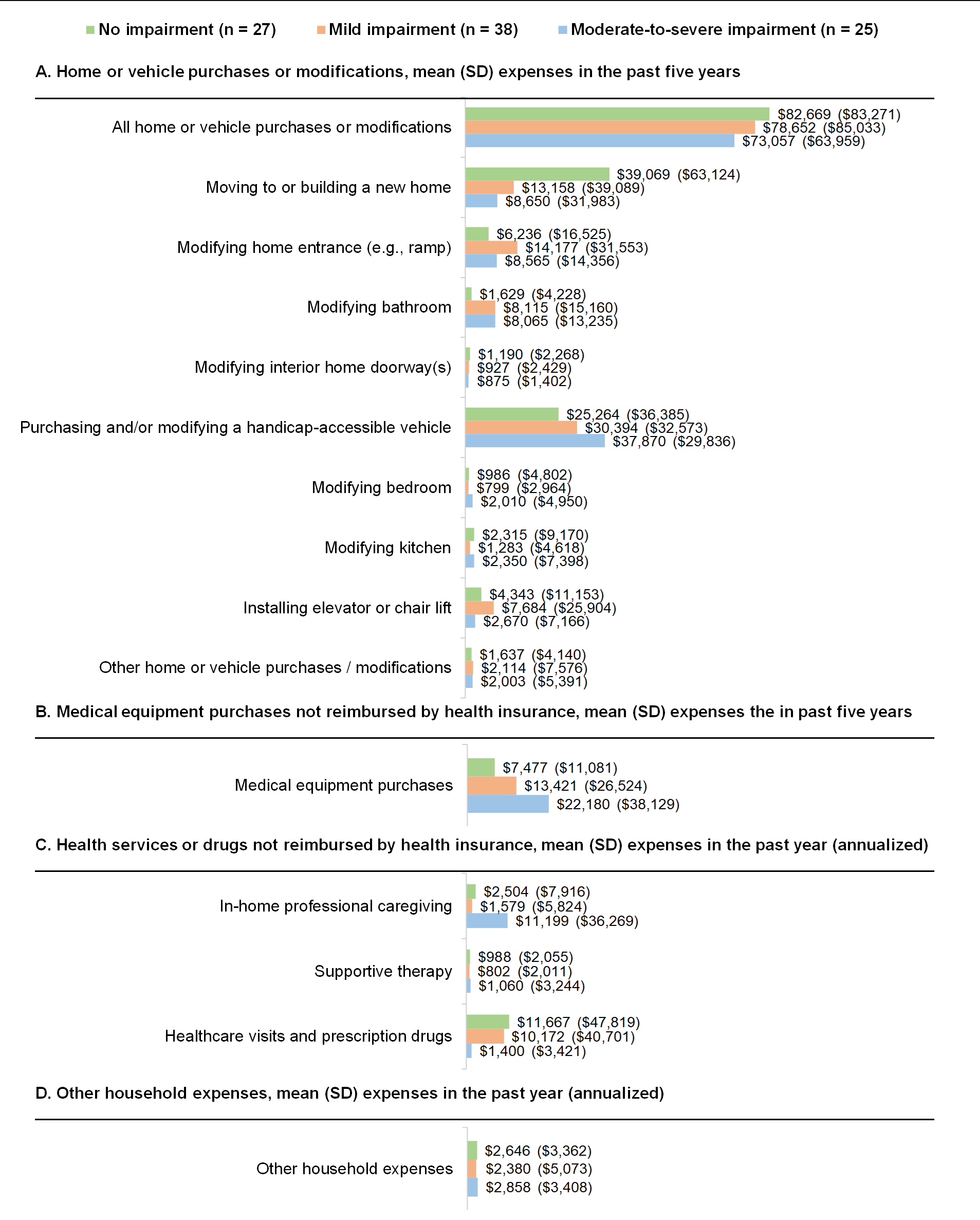

Supplement: Supplementary file 4 — Supplementary Material 4 [file 13023_2025_3794_MOESM4_ESM.png]

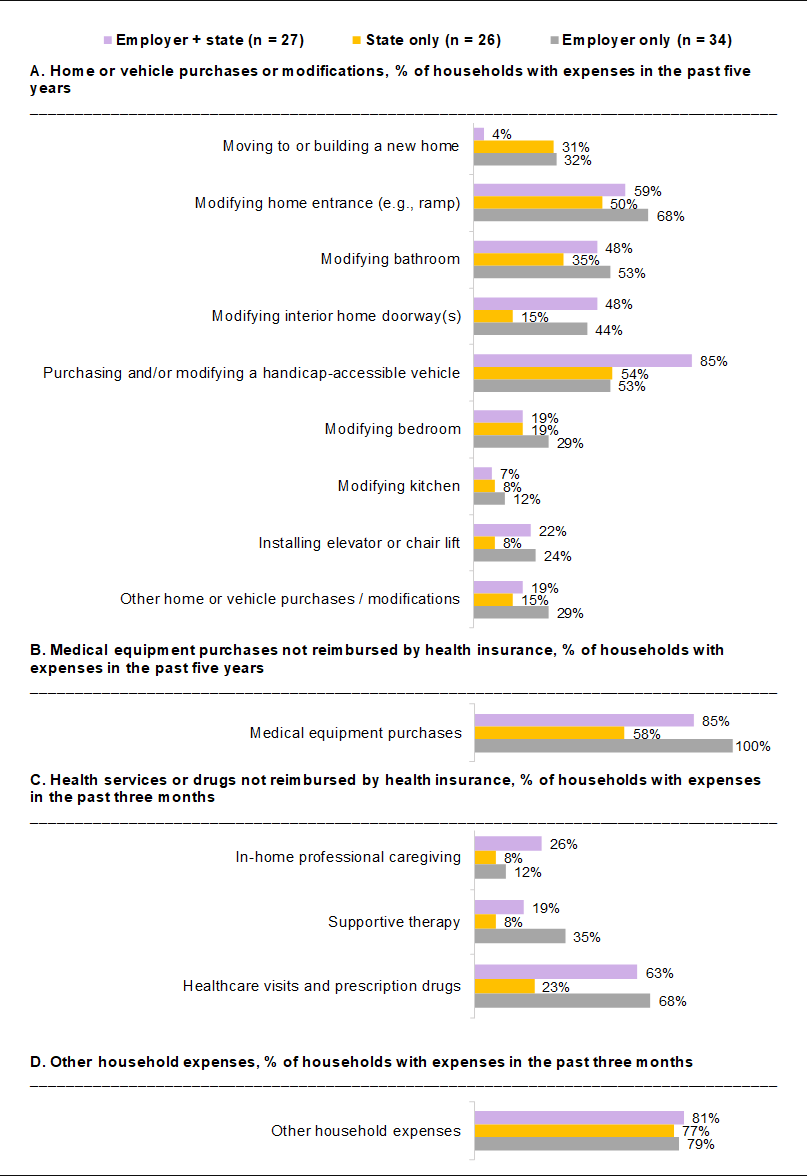

Supplement: Supplementary file 5 — Supplementary Material 5 [file 13023_2025_3794_MOESM5_ESM.png]

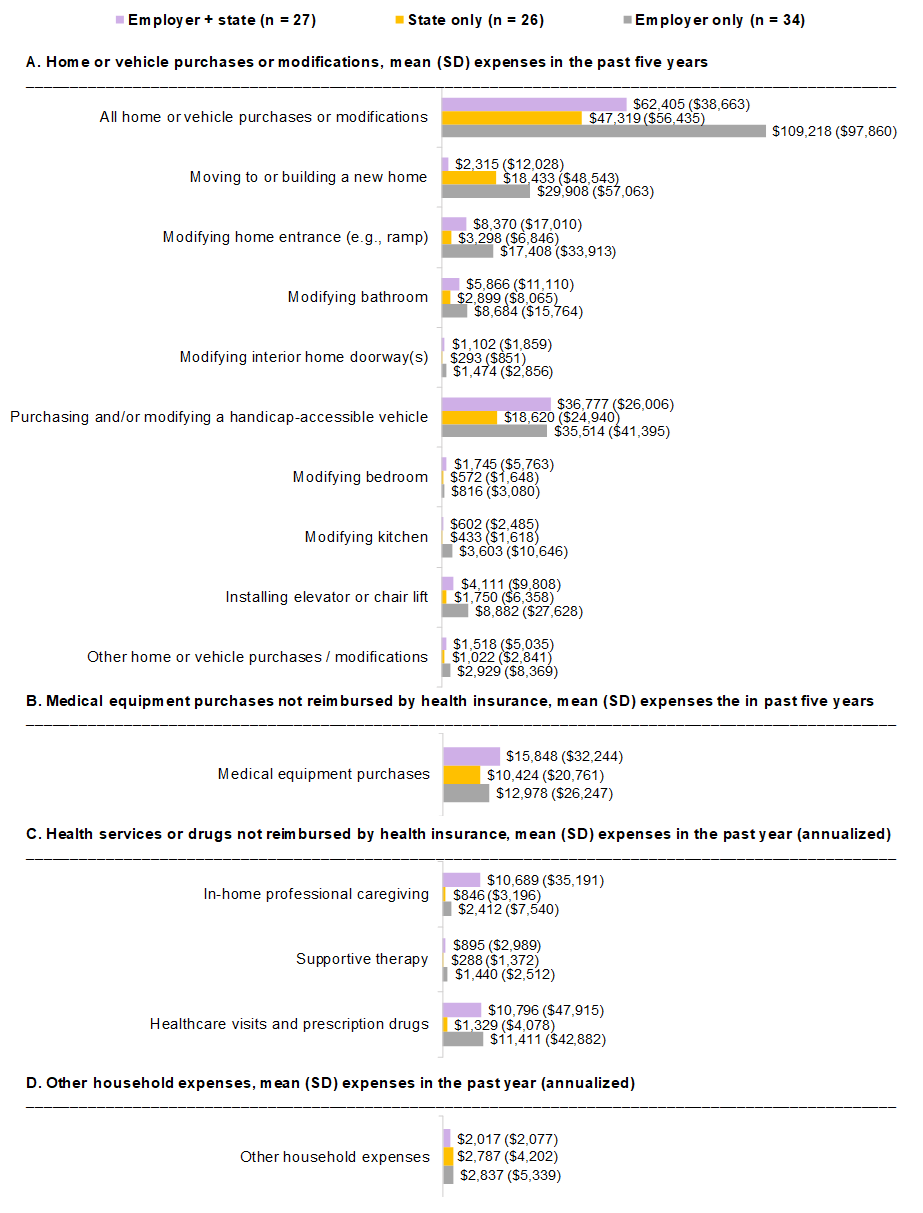

Supplement: Supplementary file 6 — Supplementary Material 6 [file 13023_2025_3794_MOESM6_ESM.png]

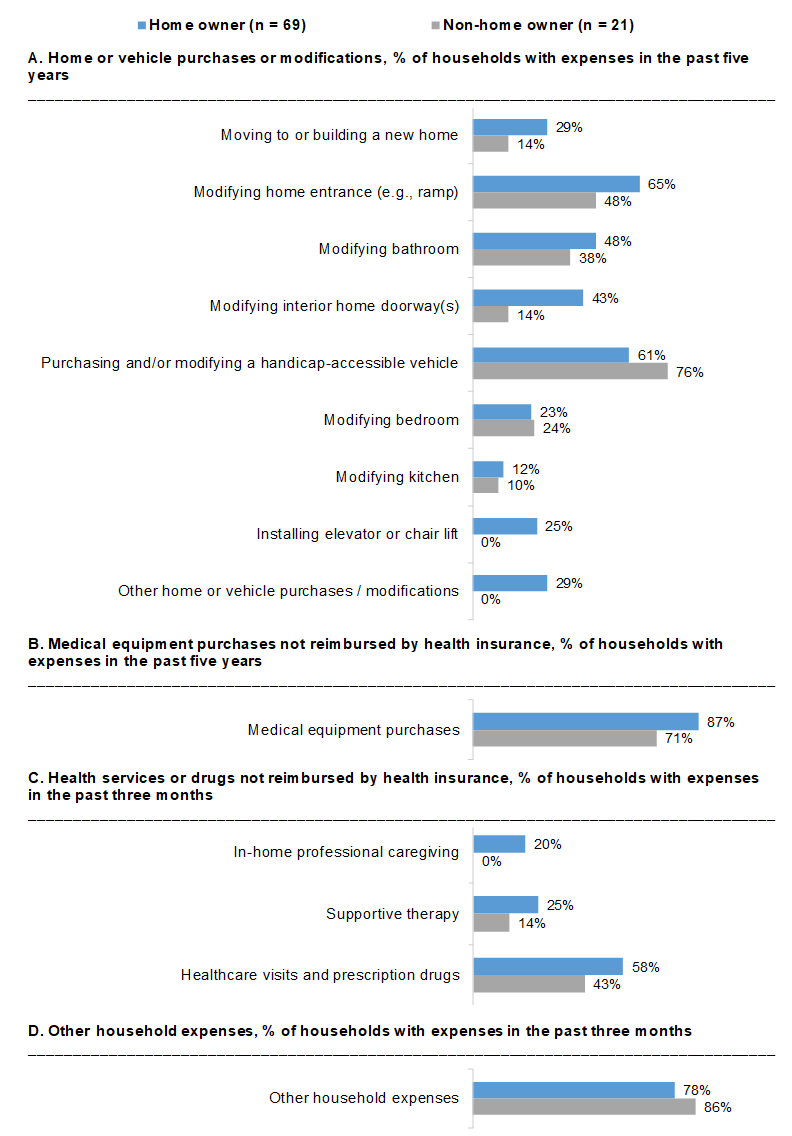

Supplement: Supplementary file 7 — Supplementary Material 7 [file 13023_2025_3794_MOESM7_ESM.png]

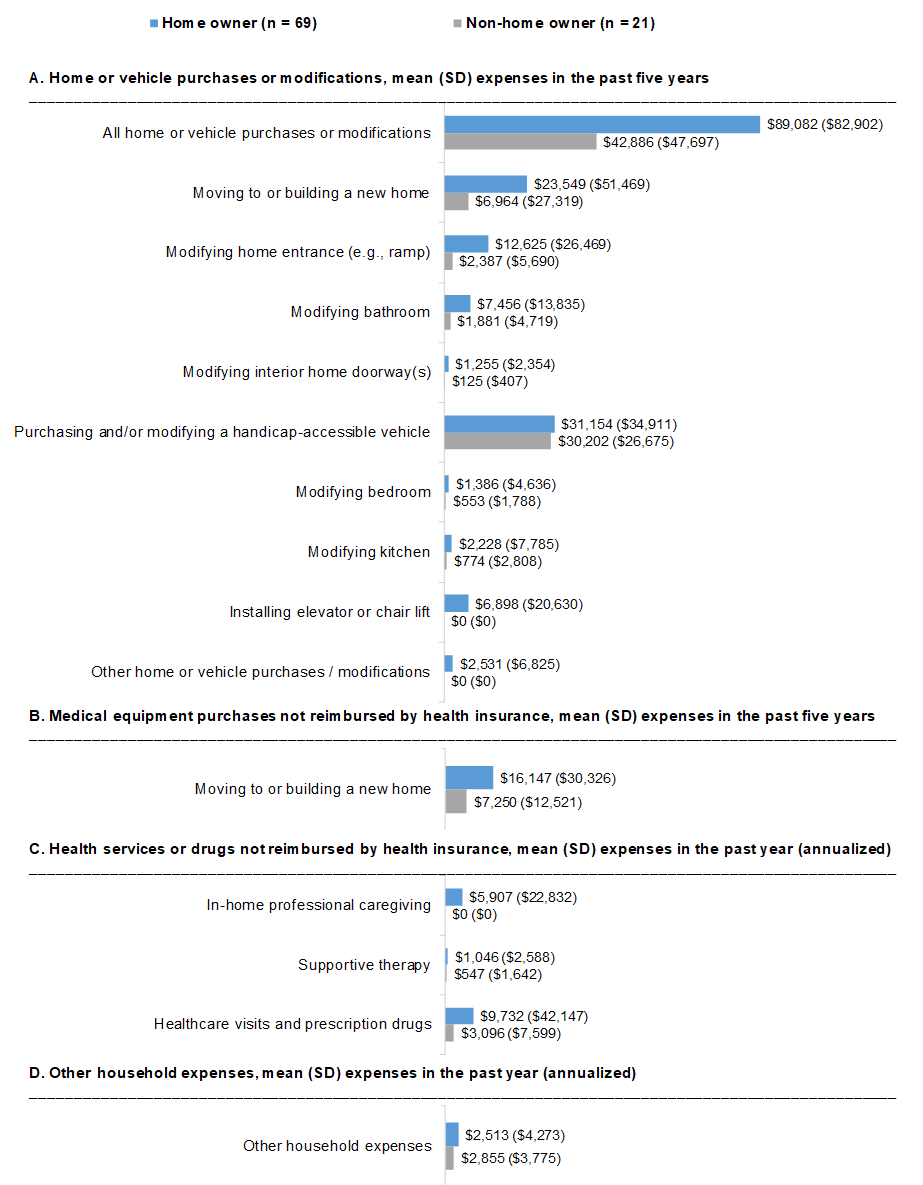

Supplement: Supplementary file 8 — Supplementary Material 8 [file 13023_2025_3794_MOESM8_ESM.png]

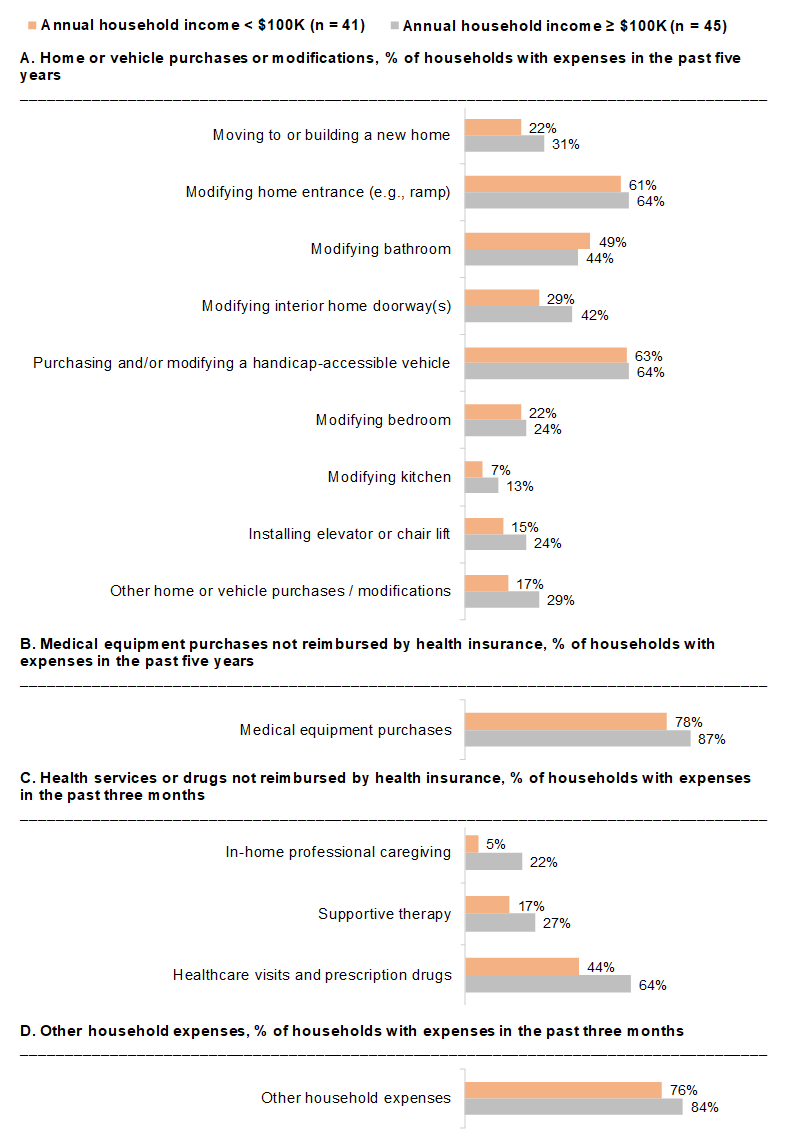

Supplement: Supplementary file 9 — Supplementary Material 9 [file 13023_2025_3794_MOESM9_ESM.png]

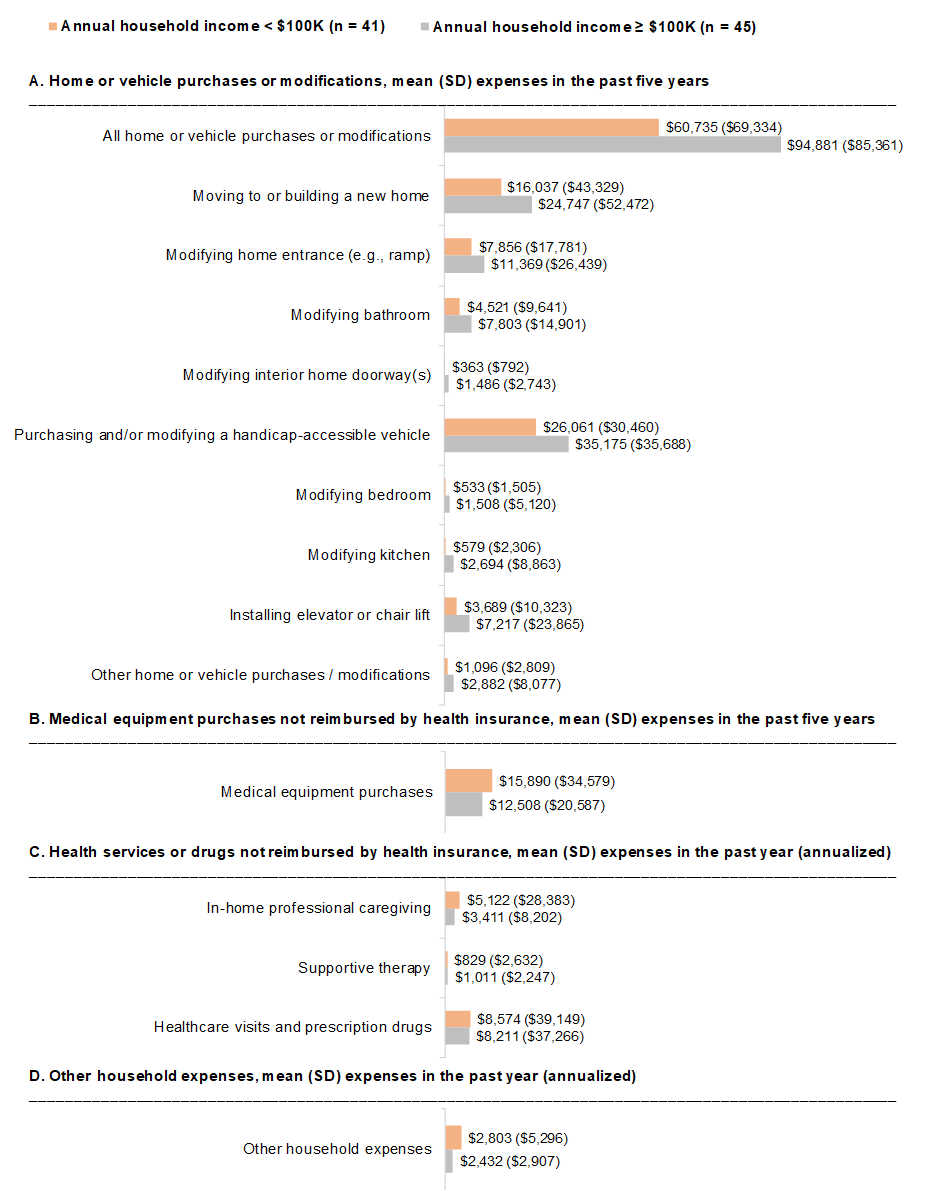

Supplement: Supplementary file 10 — Supplementary Material 10 [file 13023_2025_3794_MOESM10_ESM.png]
