# Supplementary material for: Household costs in the United States for accommodating functional impairments associated with Duchenne muscular dystrophy: results from a caregiver survey
Source: Orphanet J Rare Dis. 2025 Jun 12;20:301. doi: 10.1186/s13023-025-03794-1 (PMC12160368; doi:10.1186/s13023-025-03794-1)
Supplement: Supplementary file 11 — Supplementary Material 11 [file 13023_2025_3794_MOESM11_ESM.docx]

**Supplemental Table S1** Additional characteristics of individuals with DMD and their caregivers, and household characteristics

| **Patient demographic characteristics** | **N = 106** |
| --- | --- |
| Health insurance coverage in the past 12 months, n (%) |  |
| Medical and pharmacy coverage | 102 (96.2) |
| Medical coverage only | 3 (2.8) |
| Pharmacy coverage only | 1 (0.9) |
| Type(s) of health insurance coverage during the past 12 months, n (%)^a^ |  |
| Employer-sponsored health insurance^b^ | 72 (67.9) |
| State health insurance (e.g., Medicaid, SCHIP)^b^ | 62 (58.5) |
| Individual health insurance through a marketplace or exchange | 2 (1.9) |
| Medicare or Medi-Gap | 12 (11.3) |
| Any military healthcare (e.g., TRICARE, VA, CHAMPVA) | 3 (2.8) |
| Other^c^ | 3 (2.8) |
| **Other clinical variables** |  |
| Wheelchair/scooter use |  |
| Wheelchair/scooter used, n (%) | 85 (80.2) |
| Age, years, mean (SD)^d^ | 10.5 (2.8) |
| Number of years using a wheelchair/scooter, mean (SD)^d^ | 5.0 (4.9) |
| Diagnosed comorbidities, n (%)^a^ |  |
| ADHD | 28 (26.4) |
| Anxiety | 37 (34.9) |
| Asthma | 6 (5.7) |
| Autism spectrum disorder | 16 (15.1) |
| Depression | 15 (14.2) |
| Diabetes | 1 (0.9) |
| Epilepsy | 4 (3.8) |
| Learning disabilities | 32 (30.2) |
| Obesity or overweight | 21 (19.8) |
| Osteoporosis | 28 (26.4) |
| Scoliosis | 30 (28.3) |
| Sleep disorder | 16 (15.1) |
| None of the above | 23 (21.7) |
| **Caregiver demographic characteristics** | **N = 90** |
| Current relationship status, n (%) |  |
| Married or living with someone | 71 (78.9) |
| Widowed | 3 (3.3) |
| Divorced | 11 (12.2) |
| Single | 5 (5.6) |
| Highest level of education, n (%) |  |
| Some high school, no diploma | 1 (1.1) |
| High school graduate, diploma, or equivalent (e.g., GED) | 7 (7.8) |
| Some college or university, no degree | 17 (18.9) |
| Trade, technical, or vocational training | 8 (8.9) |
| Bachelor’s degree | 33 (36.7) |
| Master’s degree | 14 (15.6) |
| Professional degree | 3 (3.3) |
| Doctorate degree | 2 (2.2) |
| Other^e^ | 5 (5.6) |
| Current employment status, n (%) |  |
| Full-time employed (35 hours per week or more) | 36 (40.0) |
| Part-time employed | 15 (16.7) |
| Paid caregiver | 10 (11.1) |
| Homemaker | 24 (26.7) |
| Unemployed | 3 (3.3) |
| Retired | 1 (1.1) |
| Other^c^ | 1 (1.1) |
| **Household-level characteristics** | **N = 90** |
| Total combined household income last year (USD), n (%) |  |
| $0 to $9999 | 0 (0.0) |
| $10000 to $24999 | 4 (4.4) |
| $25000 to $49999 | 11 (12.2) |
| $50000 to $99999 | 26 (28.9) |
| $100000 to $149999 | 27 (30.0) |
| $150000 to $199999 | 8 (8.9) |
| $200000 to $249999 | 2 (2.2) |
| $250000 to $299999 | 3 (3.3) |
| $300000 and higher | 5 (5.6) |
| Other^c^ | 4 (4.4) |
| Region, n (%) |  |
| Northeast | 15 (16.7) |
| Midwest | 28 (31.1) |
| South | 33 (36.7) |
| West | 14 (15.6) |
| Community type, n (%)^e^ |  |
| Urban city | 11 (12.2) |
| Town or suburban area outside of a city | 52 (57.8) |
| Rural or countryside area | 27 (30.0) |
| Property ownership status, n (%) |  |
| Owned or co-owned property | 69 (76.7) |
| Rented or co-rented property | 18 (20.0) |
| Relatives’ or friends’ property | 3 (3.3) |
| Number of individuals currently living in household, mean (SD) |  |
| Children (aged 0–17) | 1.7 (1.3) |
| Adults (aged 18–64) | 2.3 (0.9) |
| Seniors (aged 65 and older) | 0.1 (0.4) |
| Number of household members working for pay in the past 12 months, n (%) |  |
| 0 | 3 (3.3) |
| 1 | 37 (41.1) |
| 2 | 44 (48.9) |
| 3 | 6 (6.7) |

ADHD, attention-deficit/hyperactivity disorder, CHAMPVA, Civilian Health and Medical Program of the Department of Veterans’ Affairs, DMD, Duchenne muscular dystrophy, GED, General Educational Development, SCHIP, State Children’s Health Insurance Program, VA, Veterans’ Affairs

^a^Participants could select multiple response categories. Therefore, percentages may sum to over 100%

^b^31 (29.2%) individuals were reported as having both employer-sponsored health insurance and state health insurance

^c^“Other” responses included options for “Other,” “Prefer not to answer,” or “Unsure”

^d^Calculated among patients reported to use a wheelchair or scooter

^e^An urban city was generally defined as having a population of at least 50,000 people. A suburban area or town was generally defined as representing a smaller community, potentially adjacent to or within the commuting distance of a city
