# Supplementary material for: Household costs in the United States for accommodating functional impairments associated with Duchenne muscular dystrophy: results from a caregiver survey
Source: Orphanet J Rare Dis. 2025 Jun 12;20:301. doi: 10.1186/s13023-025-03794-1 (PMC12160368; doi:10.1186/s13023-025-03794-1)
Supplement: Supplementary file 12 — Supplementary Material 12 [file 13023_2025_3794_MOESM12_ESM.docx]

**Supplemental Table S2** Conditional median household expenses

|  | **Conditional median^a,b^** | | |
| --- | --- | --- | --- |
|  | **Households that incurred costs, n (% of all households)** | **Conditional median (25^th^ percentile – 75^th^ percentile) costs** | |
| **Home or vehicle purchases or modifications (5-year cost)** | 90 (100) | - | |
| Moved to or built a new home | 23 (26) | $62,500 ($7500-$150,000) | |
| Modified home entrances (e.g., ramp) | 55 (61) | $3750 ($750-$17,500) | |
| Modified bathroom | 41 (46) | $7500 ($750-$17,500) | |
| Modified interior home doorway(s) | 33 (37) | $1750 ($375-$3750) | |
| Purchased and/or modified a handicap-accessible vehicle | 58 (64) | $45,000 ($25,000-$62,500) | |
| Purchased a new vehicle | 17 (19) | $62,500 ($62,500-$62,500) | |
| Purchased a used vehicle | 39 (43) | $35,000 ($25,000-$62,500) | |
| Other | 7 (8) | $375 ($375-$12,500) | |
| Modified bedroom | 21 (23) | $1750 ($375-$3750) | |
| Modified kitchen | 10 (11) | $12,500 ($7500-$25,000) | |
| Installed elevator or lift | 17 (19) | $17,500 ($3750-$35,000) | |
| Elevator | 5 (6) | $25,000 ($17,500-$35,000) | |
| Stair lift | 6 (7) | $3750 ($1750-$7500) | |
| Platform lift | 3 (3) | $25,000 ($25,000-$35,000) | |
| Pool lift | 4 (4) | $12,500 ($7500-$12,500) | |
| Ceiling track | 7 (8) | $12,500 ($3750-$25,000) | |
| Patient lift (e.g., Hoyer lift, Molift) | 6 (7) | $1750 ($1750-$1750) | |
| Other home or vehicle purchases or modifications | 20 (22) | $2750 ($1750-$7500) | |
| **Medical equipment purchases not reimbursed by health insurance (5-year cost)** | 75 (83) | $3750 ($1750-$17,500) | |
| Scooter | 18 (20) | $1750 ($750-$3750) | |
| Stroller | 7 (8) | $250 ($125-$1750) | |
| Powered wheelchair | 39 (43) | $3750 ($437-$30,000) | |
| Manual wheelchair | 25 (28) | $375 ($125-$2750) | |
| Foldable/travel wheelchair | 31 (34) | $1750 ($375-$3750) | |
| Walker/leg braces (e.g., ankle foot orthotics) | 30 (33) | $375 ($125-$1750) | |
| Safety/hospital bed(s) | 22 (24) | $750 ($125-$7500) | |
| BiPAP machine | 8 (9) | $125 ($125-$375) | |
| Cough assist machine | 25 (28) | $375 ($125-$1750) | |
| Other breathing assists | 4 (4) | $1937 ($125-$3750) | |
| Other medical equipment | 16 (18) | $750 ($750-$1750) | |
| **Health services or drugs not reimbursed by health insurance (1-year cost, annualized)** |  |  | |
| In-home professional caregiving | 14 (16) | $14,998 ($6998-$29,998) | |
| Supportive therapy | 20 (22) | $2998 ($1498-$6998) | |
| Healthcare visits and prescription drugs | 49 (54) | $1498 ($498-$6998) | |
| **Other expenses (1-year cost, annualized)** | 72 (80) | $1498 ($498-$2998) | |
| BiPAP, bilevel positive airway pressure  ^a^ 5-year costs are presented for items related to home or vehicle purchases or modifications and medical equipment purchases not reimbursed by health insurance. 1-year, annualized costs are presented for health services or drugs not reimbursed by health insurance. 1-year, annualized costs are presented for other expenses  ^b^ Calculated among households that incurred costs and provided a corresponding cost estimate | | |  |
