# Supplementary material for: Household costs in the United States for accommodating functional impairments associated with Duchenne muscular dystrophy: results from a caregiver survey
Source: Orphanet J Rare Dis. 2025 Jun 12;20:301. doi: 10.1186/s13023-025-03794-1 (PMC12160368; doi:10.1186/s13023-025-03794-1)
Supplement: Supplementary file 13 — Supplementary Material 13 [file 13023_2025_3794_MOESM13_ESM.docx]

**Supplemental Methods**

**Survey Components**

***Ambulatory status of individuals with DMD***

Survey question: which of the following statements best describes how they get around and how often they use a wheelchair or scooter?

| **Survey response** | **Category** |
| --- | --- |
| They walk all day. They may or may not use a wheelchair or scooter for long distances on special occasions (on vacation, at an amusement park or the zoo). | Ambulatory |
| They use a wheelchair or scooter some of the day on most days of the week throughout the year, but they can regularly walk down the hall in your home without holding anything. | Transitional |
| They always use a wheelchair or scooter. They may or may not be able to take a few steps by themselves. | Non-ambulatory |
| Unsure. | Uncategorized |

***Upper limb function of individuals with DMD***

Survey question: which of the following statements best describes how well they can use their hands and arms?

| **Survey response** | **Category** |
| --- | --- |
| They can raise both arms above their head at the same time, without having to make any adjustments. | No impairment |
| They have difficulty raising both arms at the same time or raising a heavy object to shoulder height or eye level. | Mild impairment |
| They cannot raise their hands above their head but can bring a full glass of water to their mouth. | Mild impairment |
| They cannot raise a full glass of water but can use their hands to hold a pen, pick up a coin, or drive power chair. | Moderate impairment |
| They are unable to pick up or hold objects. | No upper limb function |
| Unsure | Uncategorized |

**Statistical Analysis**

***Household costs***

Mean (SD) costs were calculated using two methods. First, they were averaged over all households represented in the sample, i.e., if a participant indicated that their household did not incur any costs, they were included in the denominator with a cost value of zero. In some cases, participants indicated that their household made a payment or purchase but did not know or did not provide the cost value; in these cases, households were assigned the average cost for the item as calculated among all non-missing responses. Second, for each item, the conditional mean (SD) cost was calculated, i.e., only including households that incurred costs and provided a corresponding cost estimate in the calculations.
